# Health Promotion Behaviors of Pregnant Couples in the COVID-19 Pandemic: Actor-Partner Interdependence Model

**DOI:** 10.3390/ijerph19127501

**Published:** 2022-06-19

**Authors:** Sun Hee Kim, Hee Sun Kang

**Affiliations:** 1 Department of Nursing, Chung-Ang University Hospital, Seoul 06973, Korea; sunny231845@naver.com; 2 Red Cross College of Nursing, Chung-Ang University, Seoul 06974, Korea

**Keywords:** COVID-19, pregnancy, couples, health promotion, health behavior, depression, fear, posttraumatic growth, marital adjustment

## Abstract

Pregnancy during the pandemic may be a stressful life event. This cross-sectional study aimed to identify the actor and partner effects of the fear of COVID-19, depression, posttraumatic growth, and marital adjustment on the health promotion behaviors of pregnant couples during the COVID-19 pandemic in Korea. Data were collected from 123 pregnant couples using a structured questionnaire. The data were analyzed using the Actor-Partner Interdependence Model (APIM). The actor effects of the fear of COVID-19, depression, and posttraumatic growth on the health promotion behaviors of pregnant women and their spouses were significant. Furthermore, both actor and partner effects of husbands’ marital adjustment on health promotion behaviors were significant. When carrying out nursing intervention for the health promotion of pregnant couples, programs aimed at reducing depression and improving posttraumatic growth in pregnant couples should be included. In addition, improving marital adjustment will positively affect the health promotion behaviors of pregnant couples. The findings highlight the important role of healthcare providers in assessing depressive symptoms and fear of COVID-19 in pregnant couples and providing support to promote health behaviors as part of prenatal care.

## 1. Introduction

Coronavirus disease 2019 (COVID-19), caused by severe acute respiratory syndrome coronavirus 2 (SARS-CoV-2), was first discovered in December 2019 in Wuhan, China, and subsequently, spread globally. The World Health Organization (WHO) declared COVID-19 to be pandemic on 11 March 2020 [1]. As of 5 March 2022, more than 420 million cases of COVID-19 had been confirmed globally, and more than 5.99 million deaths had been attributed to COVID-19 [2]. The COVID-19 pandemic has severely affected our daily lives and health [3,4,5]. Some people are at higher risk from contracting COVID-19, including older people, people with health conditions, and pregnant women [6]. Pregnant women are at increased risk of severe illness from COVID-19 and adverse pregnancy outcomes, including premature births, Cesarean section, intrauterine growth retardation, and low birth weight [7,8,9,10,11,12,13], indicating the importance of pregnant women’s health management during the pandemic.

Pregnant women faced difficulties in obtaining adequate antenatal care during the COVID-19 pandemic due to limited access to health care [14,15] and were reluctant to visit antenatal care due to fear of COVID-19 infection, resulting in a decrease in antenatal care visits [16]. Furthermore, pregnant women were concerned about their own and their babies’ health [17]. A study reported that pregnant couples were more likely comply with COVID-19 preventive behaviors due to a heightened fear of COVID-19 [18]. Moreover, fear of COVID-19 was positively correlated with depressive symptoms in pregnant women [19].

The COVID-19 pandemic has negatively affected mental health among both the general public and pregnant women [14,20,21,22,23,24,25]. A study comparing the levels of depressive symptoms of pregnant women before and during the pandemic also showed that the depressive symptoms during the pandemic were higher [21]. Furthermore, pregnant women’s depressive symptoms negatively affected their health promotion behaviors (HPB) [26,27].

Pregnancy during the pandemic is a stressful life event. Studies report that posttraumatic growth (PTG), which is the positive psychological change resulting from challenging life events or circumstances [28], may occur in people after a stressful life event [29,30,31,32,33]. Studies regarding PTG in perinatal women focused mainly on childbirth and adverse pregnancy outcomes, including preterm births and pregnancy loss [33,34,35,36,37,38]. There is a lack of studies on PTG in pregnant couples during the COVID-19 pandemic. In particular, studies regarding the impact of PTG in pregnant couples on their HPB during the pandemic are lacking. 

Couples are in an interdependent relationship; thus, they could influence each other’s health behaviors. A study conducted on pregnant couples reported that marital adjustment positively affected their HPB [39]. A survey conducted on couples in Spain reported that marital adjustment decreased during the COVID-19 pandemic compared to before the pandemic [40]. In addition, COVID-19 affected the lifestyles of pregnant women, causing a decrease in physical activity and quality of diet and sleep, and an increase in a sedentary lifestyle [25]. Thus, a dyadic approach may provide further insight into the HPB of pregnant couples during the pandemic. 

**Figure 1:**
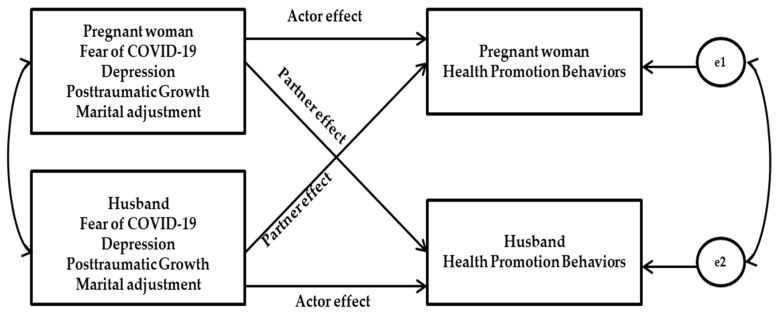
Conceptual framework of the study. Note. e1 and e2: measurement error variance.

The Actor-Partner Interdependence Model (APIM) provides a framework for examining the influence that members of a dyad have on each other [41] and is ideal for explaining spouses’ level of interdependence. The APIM estimates the actor effects (the effect of a person’s independent variable on an outcome variable) and partner effects (the effect of a partner’s independent variable on an outcome variable). Figure 1 illustrates a conceptual framework of the APIM. Understanding the factors that affect HPB among couples is essential to improve the quality of care. Therefore, this study aimed to identify the actor and partner effects of fear of COVID-19, depression, posttraumatic growth, and marital adjustment on the health promotion behaviors of pregnant couples during the COVID-19 pandemic. 

## 2. Materials and Methods

### 2.1. Study Population

This cross-sectional study was conducted with a convenient sample of 123 pregnant women and their husbands. The participants were recruited from one university-affiliated tertiary hospital and two online communities popular among pregnant women. The inclusion criteria were: (1) aged 18 years and above, (2) pregnant couple, and (3) married. The exclusion criteria were: (1) pregnant women who did not live with their husbands, and (2) those who did not agree to participate voluntarily. 

### 2.2. Measures

#### 2.2.1. Fear of COVID-19

Fear of COVID-19 was measured using the Fear of COVID-19 Scale [42], originally developed by Ahorsu et al. [20]. This scale comprises seven items rated on a 5-point Likert scale ranging from 1 (*strongly disagree*) to 5 (*strongly agree*). Total scores range from 7 to 35, with higher scores indicating greater fear of COVID-19. In this study, the Cronbach’s alpha coefficients for pregnant women and partners were 0.85 and 0.86, respectively.

#### 2.2.2. Depression

Depression symptoms were measured using the validated Korean version of Center for Epidemiologic Studies Depression Scale (CES-D) [43,44]. This scale comprises 20 items rated on a 4-point Likert scale ranging from 0 (*rarely or none of the time*) to 3 (*most or almost all the time*). Total scores range from 0 to 60, with higher scores indicating greater levels of depression. In this study, the Cronbach’s alpha coefficients for pregnant women and partners were 0.92 and 0.88, respectively.

#### 2.2.3. Post Traumatic Growth

Posttraumatic growth (PTG) was measured using the validated Korean version of PTG inventory [45], which was developed by Tedeschi and Calhoun [46]. This scale comprises 16 items rated on a 6-point Likert scale ranging from 0 (*I did not experience this as a result of my crisis*) to 5 (*I experienced this change to a very great degree as a result of my crisis*). Total scores range from 0 to 96, with higher scores indicating greater levels of PTG. In this study, the Cronbach’s alpha coefficients for pregnant women and partners were 0.82 and 0.92, respectively. 

#### 2.2.4. Marital Adjustment

Marital adjustment was measured using the validated Korean version of Revised Dyadic Adjustment Scale [47]. Spanier [48] developed the Dyadic Adjustment Scale and Busby et al. [49] revised it. This scale comprises 14 items rated on a 6-point Likert scale ranging from 0 (*always disagree/never*) to 5 (*always agree/all the time*). Total scores range from 0 to 70, with higher scores indicating greater levels of marital adjustment. In this study, the Cronbach’s alpha coefficients for pregnant women and partners were 0.86 and 0.90, respectively. 

#### 2.2.5. Health Promotion Behavior 

Health promotion behaviors was measured using the validated Korean version of Health-Promoting Lifestyle Profile, developed by Walker et al. and revised [50,51,52]. This scale comprises 26 items rated on a 4-point Likert scale ranging from 1 (*never*) to 4 (*regularly*). Total scores range from 26 to 104, with higher scores indicating greater levels of HPB. In this study, the Cronbach’s alpha coefficients for pregnant women and partners were 0.86 and 0.83, respectively. 

### 2.3. Data Collection

Data were collected from the C university-affiliated tertiary hospital in Seoul, South Korea, and two websites for pregnant women (mom cafe). The data were collected from 1 October 2021 to 1 November 2021. Participants were informed about the study’s aims and the anonymous, confidential, and voluntary nature of participation. They were also informed about their right to withdraw at any time during the study without any penalty. The self-administered survey questionnaire was administered to those who agreed to participate in the study among those who visited the antenatal clinic for their regular check-ups. Due to the COVID-19 pandemic, data were also collected online. A study recruitment notice was posted on two popular websites for pregnant women. Interested couples accessed the online (Google URL) link individually. Those who agreed and provided informed consent proceeded to complete the questionnaire. 

The questionnaire took approximately 20–30 min to be completed. In total, 125 couples (90 couples from online survey) participated in the study. Two couples from the online survey who provided incomplete answers were excluded. Data collected from 123 couples were used for analysis. The participants received a gift card worth 10,000 South Korean won (approximately 8 USD; 1000 won is equivalent to 0.8 USD) for completing the questionnaires. 

### 2.4. Data Analysis

Data were analyzed using SPSS Win version 25.0 and AMOS version 25.0 programs (IBM Corp, Armonk, NY, USA). Descriptive statistics were used to summarize the sample characteristics and the study variables. We used structural equation modeling using path analysis to test actor and partner effects of fear of COVID-19, depression, PTG, and marital adjustment on the HPB of couples. The correlations between the variables were analyzed using Pearson’s correlation coefficient. To verify the dyadic modeling, chi-square test (χ^2^), χ^2^/df, Standard Root Mean Squared Residual (SRMR), Tucker-Lewis Index (TLI), Comparative Fit Index (CFI), and Root Mean Square Error of Approximation (RMSEA) were used. In this study, a good model fit [53] was defined by the following criteria: SRMR < 0.08, RMSEA ≤ 0.06, and CFI and TLI ≥ 0.95. 

## 3. Results

### 3.1. Characteristics of Participants

A total of 123 dyads (pregnant women and their husbands) took part in this study. On average, the husbands were slightly older (34.97 ± 3.55) than their pregnant wives (33.46 ± 2.87). Most participants had a college education (83.3%). More than half of the participants reported that they had no particular religion (59.3%). More husbands (98.4%) than wives (57.7%) were employed. Most of the participants had middle to high household income (92.5%). A total of 62.6% of the participants had been married three years or longer, and the rest for less than three years (37.4%). The average duration of pregnancy was 26.6±6.5 weeks. 

### 3.2. Correlations among Study Variables

The means and standard deviations of the study variables are presented in Table 1. HPB were positively correlated with fear of COVID-19, PTG, and marital adjustment, and negatively correlated with depression among both wives and husbands (Table 1). HPB of pregnant women were positively correlated with their husbands’ HPB (*r* = 0.32, *p* < 0.001) and their marital adjustment (*r* = 0.22, *p* = 0.014). 

### 3.3. Actor-Partner Interdependence Model (APIM) Analysis

**Figure 2:**
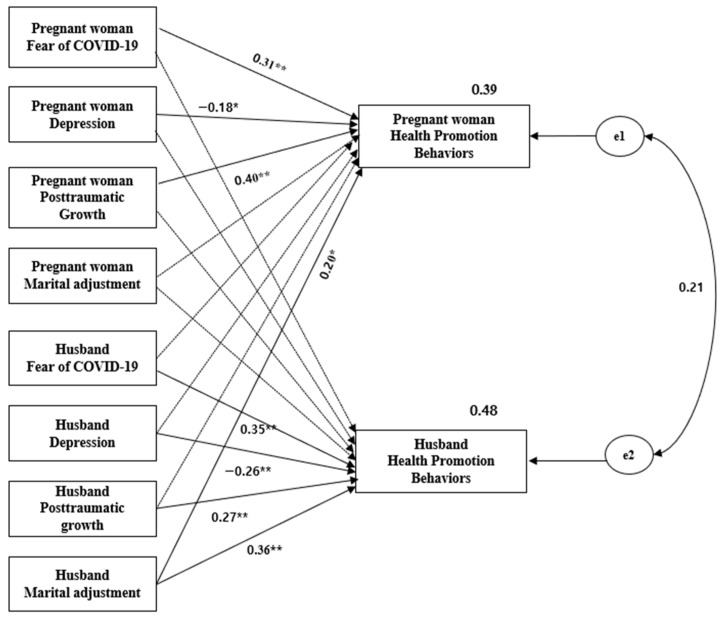
Actor and partner effects on health promotion behaviors. Note. * *p* < 0.05, ** *p* < 0.001; e1 and e2: measurement error variance.

The significance of paths in the model is shown in Figure 2.

The standardized path coefficient that accompanies each arrow in the model represents the strength of the relationship between variables. The model fit indices were as follows: χ^2^ = 0.00, df = 0, CFI = 1.00, TLI = 1.00, RMSEA = 0.00, SRMR = 0.00. The model fit indices determined that the model was a good fit for the data. As illustrated in Figure 2, all the actor effects, except the marital adjustment of pregnant women, were significant. Fear of COVID-19 had actor effects on the HPB of both pregnant women (ß = 0.31, *p* < 0.001) and their husbands (ß = 0.35, *p* < 0.001). Similarly, depression had actor effects on the HPB of both pregnant women (*β* = −0.18, *p* = 0.031) and their husbands (ß = −0.26, *p* < 0.001), and PTG had actor effects on the HPB of both pregnant women (*β* = 0.40, *p* < 0.001) and their husbands (*β* = 0.27, *p* <.001). The marital adjustment of husbands had both actor (*β* = 0.36, *p* < 0.001) and partner (*β* = 0.20, *p* = 0.013) effects on HPB. The model accounted for 39% of the variance in pregnant women’s HPB and 48% of the variance in husbands’ HPB.

## 4. Discussion

The study revealed that fear of COVID-19, depression, and PTG had actor effects on pregnant women’s and their husbands’ HPB, and husbands’ marital adjustment had actor and partner effects on HPB.

The present study identified that the fear of COVID-19 had actor effects on HPB. This result is consistent with a previous study, which reported that preventive behaviors were higher in pregnant couples with greater fear of COVID-19 than others [18]. This indicates that certain levels of fear might facilitate compliance with healthy behaviors. However, severe fear of COVID-19 could be a barrier to preventive behaviors [54]. A study with pregnant and postpartum women reported that fear and anxiety about being infected with COVID-19 negatively affected the women’s healthy diet and regular physical activities [55]. Moreover, studies reported that fear of COVID-19 was high among the general population and pregnant women [56,57] and was positively correlated with depressive symptoms [19,56]. Furthermore, pregnant women’s quality of life decreased as fear of COVID-19 increased [57]. Therefore, when providing prenatal care and education, health care providers should assess pregnant women’s fear of COVID-19 and HPB and promote mental health as a preventive strategy. 

We found that depression in couples had actor effects on HPB. This result supports a previous finding showing that depressive symptoms during pregnancy negatively affect health behaviors [27]. Studies reported that the COVID-19 pandemic negatively affected the mental health of people [21,22] and depressive symptoms were more prevalent during the COVID-19 pandemic than before [22]. In particular, depressive symptoms were higher as pregnant women felt socially isolated during the pandemic [58]. Consistent with previous studies, this study indicates the importance of preventing and alleviating depressive symptoms to encourage HPB. 

The present results show that the PTG of pregnant couples had actor effects on HPB. Depressive symptoms of women after childbirth negatively affected their PTG [38]. Thus, preventing and managing depressive symptoms may positively affect PTG, which in turn would help promote healthy behaviors in couples. Previous studies reported that stress negatively affected PTG in women who delivered premature babies [36]. This study contributes to filling the literature gap regarding the effects of pregnant couples’ PTG during the COVID-19 pandemic on their HPB. 

Our findings showed the marital adjustment of husbands had both actor and partner effects on HPB. Another study found that the actor and partner effects of marital adjustment on HPB were significant in married pregnant couples [39]. The scarcity of studies conducted on HPB of pregnant couples makes it difficult to assess the consistency of this finding. Therefore, further studies are warranted. Nevertheless, this finding indicates that the perceived marital adjustment of husbands could affect pregnant women’s HPB. A study reported that couples’ marital adjustment during pregnancy was influenced by positive dyadic coping behaviors [59]. Therefore, guiding pregnant couples to engage in positive dyadic coping could contribute to promoting their marital adjustment and HPB. Moreover, the proposed model in this study accounted for 39% of the variance in pregnant women’s and 48% of the variance in their husbands’ HPB. Therefore, we suggest further studies exploring other factors that could affect the HPB of pregnant couples. 

This study has several limitations. First, data were collected from a convenience sample that might limit the generalizability of our results. Second, the present study used a cross-sectional design, which limits determining causal relationships among the variables of interest. Longitudinal research with pregnant couples could generate a more in-depth understanding of the variables’ causality over time. Third, we used the “Fear of COVID-19 scale,” which was not validated for the Korean population, limiting the findings of the study. As of 1 November 2021, the total number of confirmed cases of COVID-19 globally and in Korea were 3,172,055 and 366,385, respectively [2]. The nationwide prevalence was approximately 709 per 100,000 people. A study by Unruh et al. [60] reported that although the challenges many countries faced during the pandemic were similar, including a shortage of healthcare workers and containment measures, there were differences in the health systems and financing. A lack of universal health coverage was considered a barrier to accessing care. In contrast, studies reported that early detection and monitoring systems using digital technology to track transmission chains of COVID-19 and the national health insurance service in Korea had a positive impact on reducing the transmission of COVID-19 [61,62]. Finally, this study was conducted with pregnant couples during the COVID-19 pandemic in Korea; as the COVID-19 pandemic situation varies from one country to another, any effort at generalization should be made with caution. It will be necessary to explore the HPB of pregnant couples in countries with differing COVID-19 protocols. Despite these limitations, we extended prior research using a dyadic approach based on the APIM to account for actor and partner effects of the study variables on HPB during the pandemic.

## 5. Conclusions

This dyadic study confirmed that fear of COVID-19, depressive symptoms, PTG, and marital adjustment affect the HPB of pregnant couples. Based on the findings of this study, it is suggested that prenatal education programs should promote PTG and marital adjustment and aim at preventing depressive symptoms in couples. 

This study showed that factors influencing HPB differed between couples. Therefore, health care personnel, educators, and others involved with designing and implementing programs to promote prenatal couples’ HPB should consider the differences in partner effects as well as considering the inhibiting and facilitating factors identified in this study. Furthermore, it is essential to be sensitive to the difficulties encountered by pregnant couples and provide support to couples experiencing poor HPB, depression, and relationship conflict. Additionally, reaching out to couples who may need support could contribute to promoting HPB in pregnant couples. Finally, this study contributes to bridging the knowledge gaps in understanding the effect of PTG in pregnant couples on their HPB during the pandemic, which has been relatively understudied in previous research.

## Figures and Tables

**Table 1:** Correlations among study variables (*N* = 123 couples).

Variable	Pregnant Women *r* (*p*)					Husbands *r* (*p*)				Total
	1 HPB	2 Fear	3 Depression	4 PTG	5 MA	6 HPB	7 Fear	8 Depression	9 MA	M ± SD
Pregnant woman										
1. HPB	1									70.46 ± 10.40
2. Fear	0.33 ***	1								24.43 ± 5.18
3. Depression	−0.21 *	0.18 *	1							17.60 ± 10.20
4. PTG	0.47 ***	0.07	−0.22 *	1						69.12 ± 3.34
5. MA	0.23 *	0.03	−0.43 ***	0.27 **	1					65.10 ± 8.54
Husband										
6. HPB	0.32 ***	0.19 *	−0.03	0.07	0.08	1				73.84 ± 14.30
7. Fear	0.06	0.20 *	0.16	−0.08	0.07	0.22 *	1			18.69 ± 5.81
8. Depression	−0.15	0.05	0.10	−0.04	−0.14	−0.39 ***	0.24 **	1		8.40 ± 6.00
9. PTG	−0.02	−0.08	−0.08	0.05	−0.08	0.38 ***	−0.11	−0.26 **	1	66.24 ± 12.64
10. MA	0.22 *	0.08	−0.00	0.05	−0.01	0.48 ***	−0.17	−0.40 ***	0.23 *	66.50 ± 9.50

* *p* < 0.05, ** *p* < 0.01, *** *p* < 0.001; Abbreviations: HPB, health promotion behaviors; Fear, fear of COVID-19; PTG, posttraumatic growth; MA, marital adjustment; M, mean; SD, standard deviation.

## Data Availability

Not applicable.
